# Diffusiophoresis and Diffusio-osmosis into a Dead-End Channel: Role of the Concentration-Dependence of Zeta Potential

**DOI:** 10.1021/acs.langmuir.2c03000

**Published:** 2023-01-28

**Authors:** Burak Akdeniz, Jeffery A. Wood, Rob G. H. Lammertink

**Affiliations:** Soft Matter, Fluidics, and Interfaces, University of Twente, MESA+ Institute for Nanotechnology, P.O. Box 217, 7500AE Enschede, The Netherlands

## Abstract

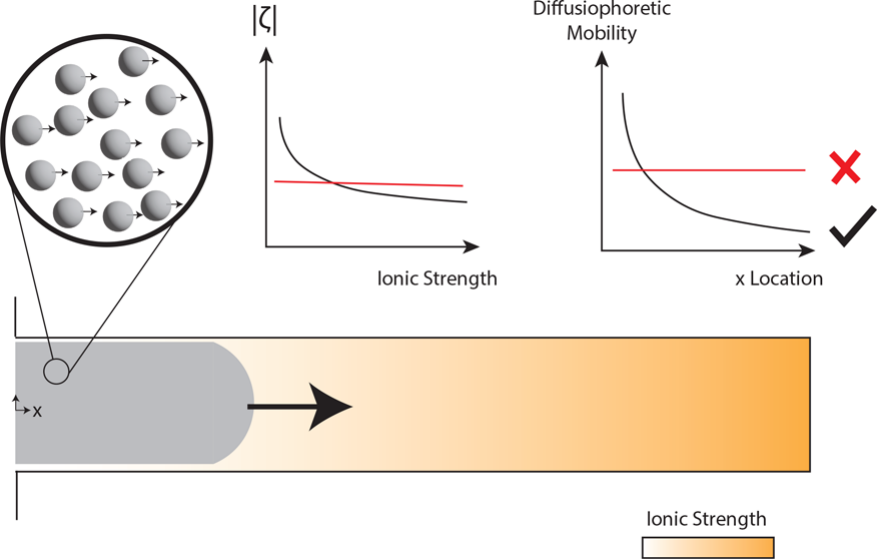

Chemically induced transport methods open up new opportunities for colloidal transport in dead-end channel geometries. Diffusiophoresis, which describes particle movement under an electrolyte concentration gradient, has previously been demonstrated in dead-end channels. The presence of solute concentration gradients in such channels induces particle motion (phoresis) and fluid flow along solid walls (osmosis). The particle velocity inside a dead-end channel is thus influenced by particle diffusiophoresis and wall diffusio-osmosis. The magnitude of phoresis and osmosis depends on the solute’s relative concentration gradient, the electrokinetic parameters of the particle and the wall, and the diffusivity contrast of cations and anions. Although it is known that some of those parameters are affected by electrolyte concentration, e.g., zeta potential, research to date often interprets results using averaged and constant zeta potential values. In this work, we demonstrate that concentration-dependent zeta potentials are essential when the zeta potential strongly depends on electrolyte concentration for correctly describing the particle transport inside dead-end channels. Simulations including concentration-dependent zeta potentials for the particle and wall matched with experimental observations, whereas simulations using constant, averaged zeta potentials failed to capture particle dynamics. These results contribute to the fundamental understanding of diffusiophoresis and the diffusio-osmosis process.

## Introduction

The interaction between a solid surface and a solute enables diffusiophoresis and diffusio-osmosis, particle or fluid displacement driven by the solute’s concentration gradient. The particle movement originates from the near surface fluid flow (osmosis) in the opposite direction of the particle movement due to a solute concentration gradient. The interaction strength between the solute and solid determines the fluid flow direction and velocity for a given concentration gradient. Derjaguin and co-workers theoretically described the concepts of diffusiophoresis and diffusio-osmosis over 70 years ago.^1−4^

Electrolyte concentration gradient-driven diffusiophoresis and diffusio-osmosis originate from two underlying mechanisms: chemiphoresis and electrophoresis.^5,6^ For chemiphoresis, the solute gradient creates a gradient in osmotic pressure within the interaction layer due to differences in the local concentration. In the case of electrophoresis, an induced electrical field, which is formed due to ion diffusivity contrast, drives the motion electrokinetically. These two contributions result in three essential parameters to determining the theoretical electrolyte diffusiophoretic velocity:^5,7^ (1) relative solute concentration gradient (∇ln(*c*)), (2) zeta potential of the particle surface (ζ), and (3) contrast between the diffusion coefficients of electrolyte ions . Additionally, physical properties of the environment such as temperature, viscosity, and dielectric constant can affect the diffusiophoretic/diffusio-osmotic velocity. These three essential contributors determine the theoretical diffusiophoretic velocity when certain assumptions are valid, i.e., a thin double layer compared to the particle size and Z:Z electrolyte. The effect of nonzero double-layer thickness on diffusiophoretic velocity has previously been analyzed theoretically^5,8,9^ and experimentally.^10^ This leads to additional parameters including the Debye length (κ^–1^) and particle radius (*a*). It was previously shown that the nonzero double layer thickness decreases the theoretical diffusiophoretic velocity, specifically in dilute electrolyte concentration regimes for negative zeta potentials.^8,11,12^ Moreover, the diffusiophoretic velocity has been theoretically analyzed considering charge regulation terms (surface charge density, zeta potential, and charge regulation coefficient), and the expression for diffusiophoretic velocity was only slightly affected for κ*a* = 1 and unaffected for κ*a* = 0 and 10.^13^ Keh and Li^13^ also highlighted the importance of the zeta potential dependence on charge regulation and electrolyte concentration.

To date, in most of the studies on diffusiophoresis and diffusio-osmosis, the zeta potential has been assumed constant when performing theoretical predictions or analyzing experimental data, and this is a reasonable assumption when the zeta potential is nearly constant within the experimental range.^14^ However, in many cases, the ion concentration^15^ or pH^14,16^ varies, and the zeta potential needs to be measured under appropriate experimental conditions since it is influenced by ionic strength, pH, electrolyte valence, and temperature.^17,18^ For example, the zeta potential for PDMS is significantly affected by salt concentration;^19^ it has been estimated at −60 mV for 10 mM KCl and −120 mV for 0.05 mM KCl, which would result in a very substantial change in the diffusio-osmotic behavior. A charge regulation model has recently been used to predict the variation of zeta potential (and its effect upon diffusiophoresis) for the pH experimental variation,^16^ and here we study the effect of varying electrolyte concentration on zeta potential during the course of diffusiophoresis into a dead-end channel.

Interest in diffusiophoresis and diffusio-osmosis phenomena with the advancement in microfluidics led to improvements in the experimental approaches to study these surface-driven phenomena. Previous experimental analysis has been conducted based on coflow,^20^ permeable walls,^21^ open channels,^22^ dead-end channels,^10,15,23^ microinjection,^24,25^ H-shaped cells,^26^ or circular hydrogel sources^27^ and were recently highlighted in several review papers.^28−30^ Moreover, those experimental structures can be used for application areas such as particle focusing,^14,20^ sorting,^31^ patterning,^32,33^ and particle/wall zeta potential measurement.^15^

The dead-end channel design, shown schematically in Figure 2A, provides an experimental platform to study diffusiophoresis and diffusio-osmosis simultaneously. Kar et al.^23^ showed that the salt gradients inside a dead-end channel generated convective flows that pressure-driven mechanisms cannot achieve in such channels. Battat et al.^34^ showed that particles were entrained by a dead-end channel when a salt concentration gradient was established inside this dead-end channel. Shin et al.^10^ demonstrated sized-based particle sorting and discussed the finite double-layer thickness effect. Gupta et al.^11^ calculated diffusiophoretic mobility under constant potential and constant charge boundary conditions for different ionic concentrations for a dead-end channel. Multi-ion and multivalence diffusiophoretic velocity has been described^35,36^ and demonstrated in dead-end channel systems to understand diffusiophoresis and diffusio-osmotic velocities for mixtures.^37−39^ Furthermore, Ault et al.^40^ showed numerical and theoretical predictions for the diffusiophoretic motion of suspended colloids in 1-D solute gradients. In follow-up work, the same group^41^ further developed 1-D and 2-D analytical expressions for the fluid, solute, and particle dynamics under diffusiophoresis/diffusio-osmosis in dead-end channels. Recently, the 3-D flow field was analyzed for different dead-end channel dimensions by Alessio et al.^42^ The ionic concentration effect on diffusiophoresis and diffusio-osmosis has been investigated by assuming constant zeta potential,^10,14,15,23,25,26^ constant surface charge,^11^ and considering surface charge regulation models.^13,16,43^ Here, we propose to include zeta potential values in diffusiophoresis and diffusio-osmosis velocity calculations by considering local ionic concentration to predict the particle transport into a dead-end channel. Moreover, previous studies experimentally analyzed particle dynamics to understand the parameters and flow patterns based on the particle distribution across the dead-end channel.^15,38^

In this work, we investigated the particle dynamics in dead-end channels experimentally and theoretically. We characterized the particle movement inside the dead-end channel using modified particle tracking analysis and identified the transient particle transport experimentally. We measured the zeta potential of particles and the wall over the range of ion concentrations predicted by simulations. Moreover, we simulated the flow, solute, and particle profiles in 3-D based on unsteady Stokes and convection-diffusion equations. Using these simulations, we compared our experimental observations and explored the influence of concentration-dependent zeta potentials on diffusiophoresis and osmosis.

## Theory

**Figure 1:**
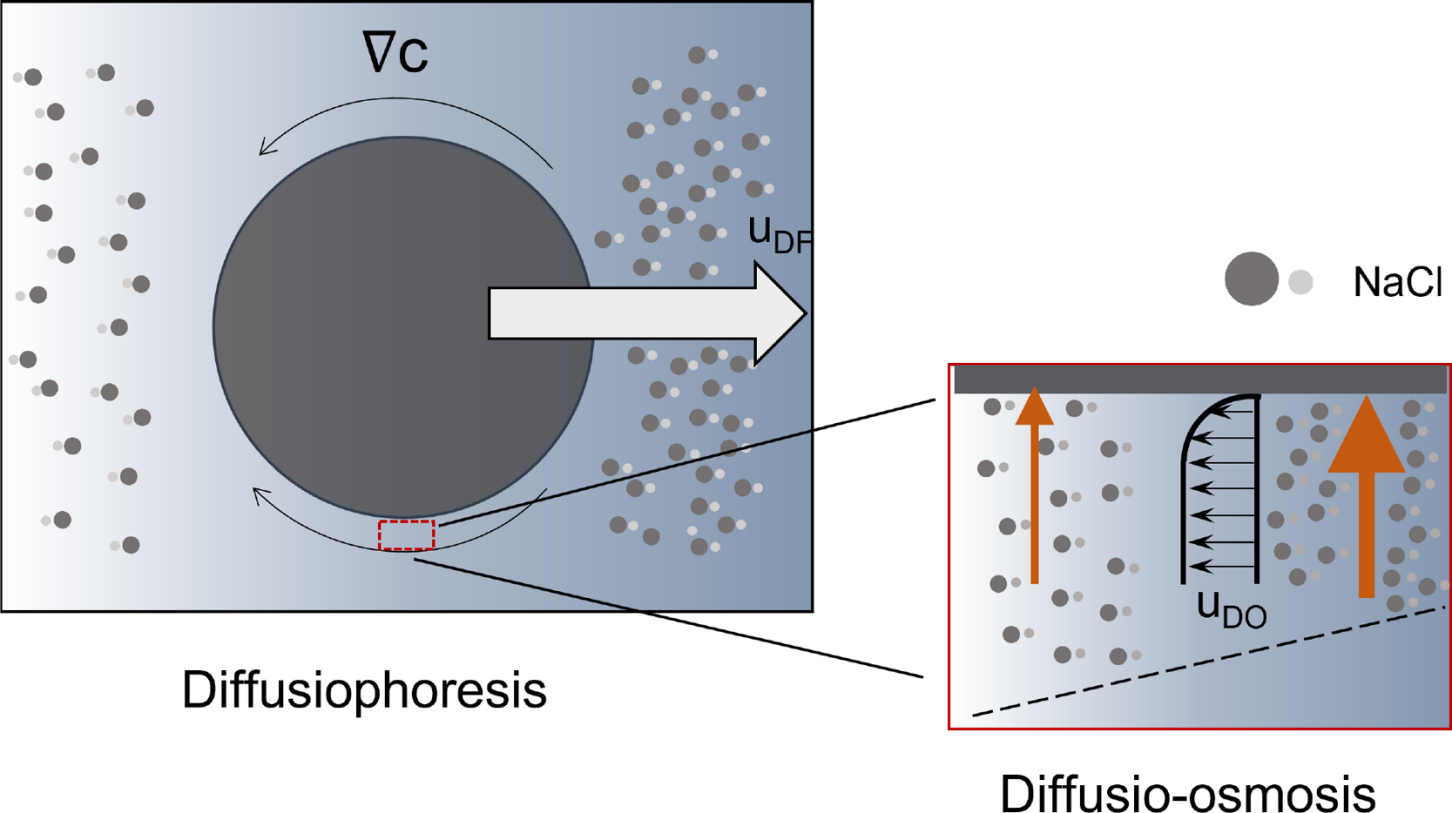
Schematics of diffusiophoresis and diffusio-osmosis phenomena. Particles migrate (diffusiophoresis) due to an electrolyte concentration gradient. The origin of the particle movement is the diffusio-osmosis at the particle surface. Diffusio-osmosis concerns the fluid movement along a surface due to a concentration gradient.

Diffusiophoresis and diffusio-osmosis are interfacial transport phenomena (Figure 1). Diffusiophoresis concerns particle movement generated by a solute concentration gradient, whereas diffusio-osmosis describes the fluid flow relative to a surface. The diffusio-osmotic and diffusiophoretic velocities are described for nonionic^44^ and ionic cases.^5^ For the electrolyte case, the concentration gradient near the surface causes a hydrostatic pressure imbalance and an electrostatic stress, which combined creates advective transport.

In the velocity description of the ionic diffusio-osmosis,^5^ the fluid inside the double layer is described by the Stokes equation, which includes the electrostatic body force term. This term is obtained by considering a Boltzmann distribution for ions combined with the electric field. When the Debye length is much less than the distance over which concentration varies appreciably,^5^ the diffusio-osmotic velocity after solving the Stokes equation reads as
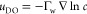
1where Γ_w_ is the diffusio-osmotic mobility. The particle diffusiophoretic and the wall diffusio-osmotic mobilities are denoted as Γ_p_ and Γ_w_, respectively. The magnitude of the diffusio-osmotic velocity is equal to the diffusiophoretic velocity when the Debye length is negligibly small compared to the particle diameter (κ*a* → ∞), but they are opposite in sign (*u*_DO_ = −*u*_DF_).^5^ Diffusio-osmosis is the fluid movement relative to the solid body (in our case it is the wall), whereas diffusiophoresis is the solid particle movement relative to the fluid. The particle diffusiophoretic mobility is different from the diffusio-osmotic mobility when the particle size is comparable with or smaller than the Debye length (κ*a* ≤ 1). The impact of Debye length on particle velocity was analyzed previously.^5,10,45^ In our work, the Debye length does not influence the particle velocity since our Debye length is much smaller than the particle diameter for the range of salt concentrations found in the dead-end channel over the time scale of experiments. The diffusiophoretic mobility term then reads as
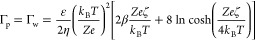
2where  quantifies the diffusivity contrast between the cation and anion (where *D*_+_ = 1.33 × 10^–9^ m^2^/s for Na^+^ and *D*_–_ = 2.03 × 10^–9^ m^2^/s for Cl^–^), ζ is the particle or wall zeta potential, ε is the medium permittivity, η is the medium viscosity (=0.001 Pa·s), *k*_B_ is the Boltzmann constant, *T* is the medium absolute temperature (=293 K), *e* is the elementary charge, and *Z* is the valence of the solute (*Z* = *Z*_Na^+^_ = −*Z*_Cl^–^_ = 1).

Electrophoresis and chemiphoresis are the two contributions to diffusiophoretic transport. Those terms are mathematically expressed as the first and second terms, respectively, of eq 2. Electrophoresis originates from the diffusivity difference between anions and cations (β), which induces a diffusion potential. This induced potential then drives the transport electrokinetically. Chemiphoresis, on the other hand, originates from the osmotic pressure gradient inside the solute–wall interaction layer. The contributions of these two terms determine the diffusio-osmotic or diffusiophoretic mobility. The magnitude of those contributions depends on the interaction strength between a solute and a surface. The signs of ζ and β determine the electrophoresis direction—toward either the high or low solute concentration side—whereas chemiphoresis is always toward the high solute concentration.^5^

### Numerical Modeling

We developed a model in 3-D to predict and understand the diffusiophoresis and diffusio-osmosis effect on particle movement within a dead-end channel. Inside the dead-end channel, a solute concentration gradient evolves. We solved the momentum transport equation for fluid and mass transfer equations for the solute and particles.

Fluid flow is governed by the unsteady Stokes (eq 3) and fluid continuity (eq 4) equations. The nonlinear inertial term is neglected due to the low Reynolds number (*Re* ≪ 1), and incompressible fluid was assumed.
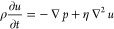
3
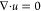
4where ρ is the fluid density, η is the fluid viscosity, and *p* is the pressure.

A wall slip velocity was assumed on the dead-end channel walls due to diffusio-osmotic flow (see below).

The convection-diffusion equation is employed for calculating the solute concentration distribution.
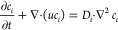
5where *D*_*i*_ is the ambipolar diffusion coefficient  (where *D*_*i*_ = 1.61 × 10^–9^ m^2^/s). For the 1-D domain, the convection term is ignored and the analytical solution for unsteady diffusion is used.^10^

The convective-diffusion equation was also employed for the particle distribution in the dead-end channel. This approach assumes particle transport based only on diffusion and convection, which we describe below. The other interactions like particle–particle or particle–wall are neglected.
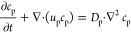
6where *D*_p_ is the particle diffusion coefficient, which is estimated using the Stokes–Einstein equation . *u*_p_ is obtained as the summation of particle diffusiophoresis (*u*_DF_ = Γ_p_∇ ln *c* from eq 1 and eq 2) and the fluid flow (*u* from eq 3 and eq 4).

### Boundary and Initial Conditions

**Figure 2:**
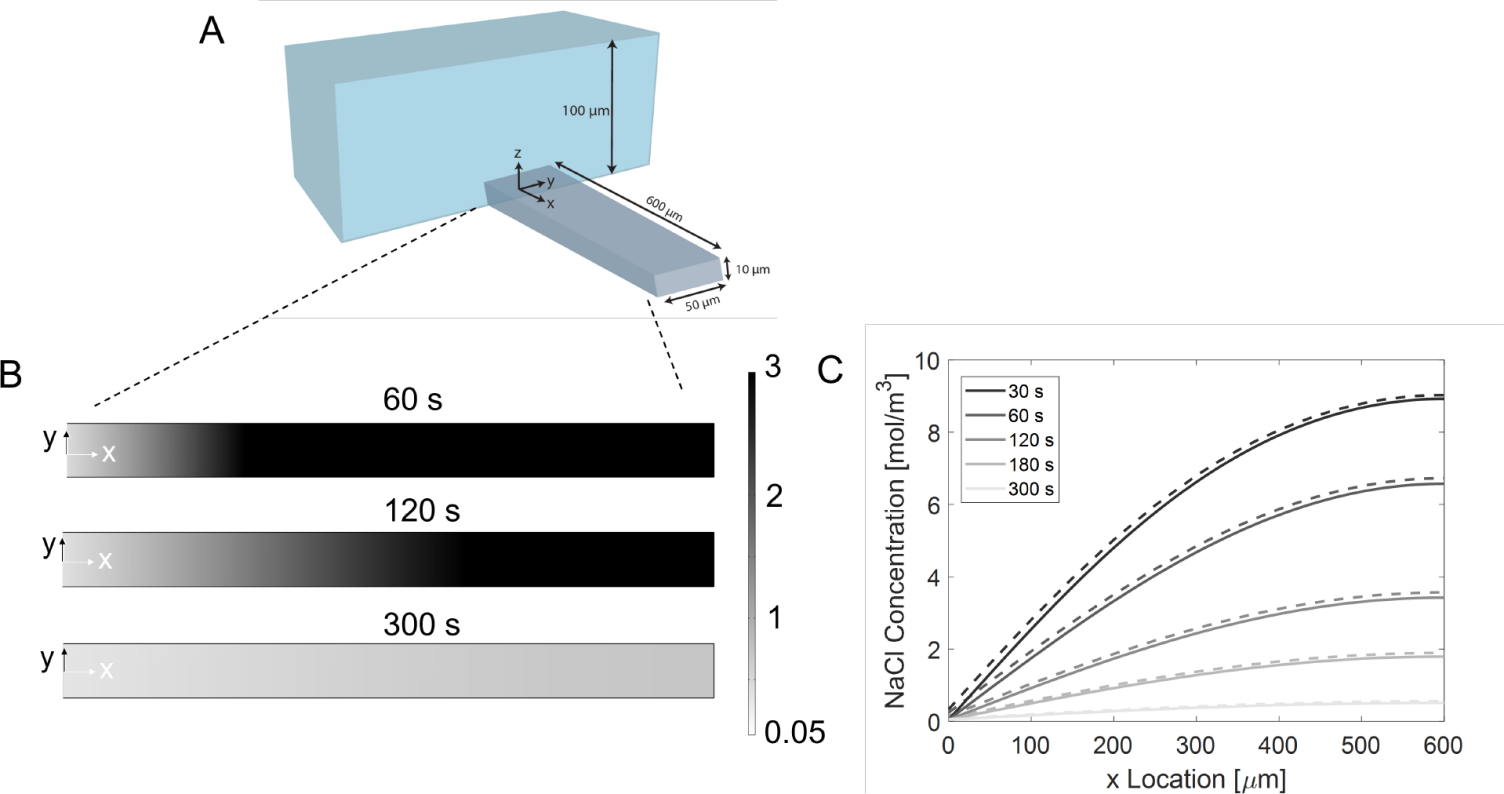
(A) The schematic representation of the dead-end channel system with dimensions. (B) NaCl concentration profile (*xy* plane at *z* = 5 μm) inside the channel from 3-D simulations for *t* = 60 s, *t* = 120 s, and *t* = 300 s. (C) 1-D diffusion equation concentration profile results are shown as solid lines. The dashed lines show the averaged concentration profile when the convective flow is also considered in the 3-D system. An ambipolar diffusion coefficient (1.61 × 10^–9^ m^2^/s) was used for both cases.

The main channel has an inlet and an outlet (Figure 2 A). The inlet velocity in the numerical model is set as 280 μm/s (*y*-*z* plane). The pressure at the outlet boundary is set to 0. Furthermore, all walls are considered impermeable. We defined an effective wall slip velocity given by the diffusio-osmotic velocity at all dead-end channel walls:
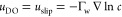
7

This diffusio-osmosis at the dead-end channel walls contributes to the convective flow within the channel (in addition to the influence of the flow by the main channel). We selected the initial conditions according to the experimental case. The initial concentration inside the dead-end channel is 10 mM NaCl. The main channel and its inlet concentration are set to 0.05 mM NaCl. The initial concentration value of particles inside the dead-end channel is 0. For the main channel and the inlet, the particle concentration is set to 1.

The above equations were solved using finite element analysis in COMSOL Multiphysics V.6.0. The momentum and convection-diffusion equations were computed with a time-dependent solver. P2+P1 discretization (second-order elements for velocity and first-order elements for pressure) was used to solve the Stokes equation. The mass transport equation is solved using second-order Lagrange elements to compute the concentration field. We assessed mesh independency by checking the concentration and velocity profiles with successive mesh refinement until profiles became constant with an increasing number of mesh elements.

## Materials and Methods

### Materials

To manufacture the polydimethylsiloxane (PDMS) devices, we used the prepolymer RTV-615 A (Permacol B.V, Ede, The Netherlands, 1020 kg/m^3^, 4300 mPa s) and the curing agent RTV-615 B (Permacol B.V, Ede, The Netherlands, 990 g/m^3^, 800 mPa s). Sodium chloride (NaCl) (99.96%) was purchased from AkzoNobel (The Netherlands). Polystyrene with rhodamine–PEG group particles, PS-RhB-PEG-Fi70–1, 1.09 ± 0.04 μm (2.5 wt %, abs/em = 560/584 nm) was purchased from Microparticles GMBH (Berlin, Germany), and FluoSpheres carboxylate, 1.00 ± 0.03 μm (2 wt %, abs/em = 580/605 nm), was purchased from Thermo Fischer (United States).

### Microfluidic Device Preparation

Two Si-wafer molds were prepared for the microfluidic device preparation: one flat Si wafer and one Si-wafer with the dead-end channel structure (positive). The dead-end channel structure was created by polydimethylsiloxane (PDMS) replication from this patterned Si wafer. This was done by mixing a prepolymer (RTV-615 A) and curing agent (RTV-615 B) in a 10:1.5 ratio. This mixture was blended for at least 5 min to get a uniform mixture and then placed in a desiccator to degas for at least half an hour. Next, it was poured onto the two Si-wafer molds and degassed again to remove all bubbles. The PDMS was cured for 4 h at 80 °C in an oven.

The flat and structured PDMS were bonded by first activating their surfaces using a Femto plasma cleaner (Diener Electronic GmbH, Ebhausen, Germany) and O_2_ plasma, for 12 s at 100 W. The main channel is 600 μm wide and 100 μm high. The dead-end channel has 50 μm width (W), 10 μm height (H), and 600 μm length (Figure 2A). The prepared microfluidic devices were soaked underwater prior to experiments to reduce the water permeation through the PDMS walls.^46^

### Experimental Protocol

A pressure-driven microfluidic pump (Fluigent MFCZ-EZ, France) was connected to the PDMS based microfluidic device and used to fill the dead-end channel with the desired solution. After filling the dead-end channel, an air bubble is passed through to empty the main channel while leaving the original solution inside the dead-end channel. Meanwhile, a particle suspension was sonicated for at least 5 min in ElmaSonic P (Elma Schmidbauer GmbH, Singen, Germany). Afterward, the particle suspension is passed through the main channel by a syringe pump (Harvard Apparatus, PHD-Ultra, Massachusetts, United States) using a 250 μL glass syringe (Hamilton, 1725RN Syringe, Nevada, United States). To minimize the particle–particle interactions and be able to track individual particles, the particle concentration was set to 0.01% w/v for PS-carboxylate and 0.05% w/v for PS–PEG. An inverted microscope (Zeiss Avio Observer. Z1, Carl-Zeiss, Jena, Germany) was employed with a 20×f/0.4 objective (depth of field is 5.8 μm, Zeiss LD Plan-Neofluar, Carl-Zeiss) and a 20HE (Carl-Zeiss, Jena, Germany) filter. The particle movement in the dead-end channel was captured by a CCD camera (Hamamatsu, Japan) with 1376 × 1040 pixels mounted in the inverted microscope. The images are sequentially captured for 6 min at 10 frames per second (fps).

### Characterization of Particle Movement

The set of images was analyzed in ImageJ, open-source image analysis software. To visualize and track the particles, the TrackMate v.4.0.1 program was used.^47^ The Laplacian of Gaussian detector feature was used for particle spot selection, and spots were connected using the linear assignment problem (LAP) tracker method, embedded inside the TrackMate program. Then, filters were applied to remove the particles stacked on the device matrix. The position value of each trajectory was further analyzed in MATLAB 2021 (Mathworks, California, United States), and the velocity of each trajectory was determined. For the 2-D velocity mapping, the dead-end channel was divided into 10 μm (through *x* direction) × 4 μm (through *y* direction) windows, in which velocity values from the particle spots were averaged. For the 1-D velocity mapping, the dead-end channel was divided into 10 μm segments in the *x* direction.

### Zeta Potential and Streaming Potential Characterization

Particle suspensions were prepared in NaCl concentrations, and the particle concentration was kept constant in the zeta potential measurement (0.005% w/v) to ensure that measurements were in the dilute regime. Electrophoretic mobility was measured using a Zetasizer Nano-ZS (Malvern Panalytical B. V., Almelo, The Netherlands) device. Henry’s function (*f*(κ*a*)) was used to correct the electrophoretic mobility  to account for particle size and salt concentration (κ*a*)^48^ with *f*(κ*a* → 0) = 1 and *f*(κ*a* → ∞) = 1.5. For intermediate salt concentrations, Henry’s function was approximated by Swan and Furst^49^ as
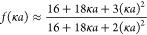
8where *a* is the radius of the particle and κ is the inverse Debye length. The zeta potential of the used particles is given in the Supporting Information Figure S1.

The streaming potential measurements were performed with PDMS flat sheets with an electrokinetic analyzer, the SurPass I (Anton Paar, Graz, Austria). An adjustable gap cell was set at 120 μm, and a 0.1–10 mM NaCl solution was used in the measurement. The streaming current value was used to estimate the zeta potential by the Helmholtz–Smoluchowski (HS) equation, eq 9.
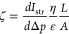
9where *dI*_str_/*d*Δ_p_ is the slope of the streaming current versus pressure data, η is the electrolyte viscosity, ε is the dielectric constant of the fluid, *L* is the length of the channel, and *A* is the cross-section of the channel. This equation applies when the surface is flat, the Debye layer is thin compared to the distance between the PDMS walls, and surface conduction can be neglected. The zeta potential of PDMS is given in Supporting Information Figure S3.

## Results and Discussion

### Solute Diffusion

The transient sodium chloride (NaCl) concentration profile in the dead-end channel (Figure 2A) was theoretically estimated by considering a 1-D domain (only the *x* direction) and a 3-D domain (Figure 2B,C). Both 1-D and 3-D show very similar NaCl concentration profile development, and only <0.5 mM salt is left inside the channel after a few minutes (≈ 300 s). The transient solute concentration gradient leads to particle diffusiophoresis and wall diffusio-osmosis. Moreover, the local solute concentration also affects electrokinetic parameters like particle and wall zeta potential.

There is a slight discrepancy (<5%) between the two concentration profiles, which is negligible beyond ∼5 μm into the dead-end channel. The difference between the 1-D and 3-D profiles increases near the dead-end channel entrance as the convective flow of the main channel is not taken into account in the 1-D case. This was included in the 3-D calculation, where the convective flow further causes concentration changes in the *y*–*z* plane at the dead-end channel entrance (see Supporting Information Figure S5). The Péclet number (*Pe* = *uW*/*D*_*i*_ based on channel width (*W*)) is <1 after ∼5–10 μm. This also indicates that the convective flow further into the channel, driven by diffusio osmosis by the walls, was low and did not cause significant ionic concentration change in the *y*–*z* direction. Nevertheless, this convective flow can strongly influence particle transport since the particle Péclet number (*Pe*_p_ = *uh*/*D*_p_ based on channel half height (*h* = *H*/2 = 5 μm) and particle diffusivity (*D*_p_ = *k*_B_*T*/6πη*a* ≈ 4 × 10^–13^ m^2^/s) in Table S1) is 50–500.

### Particle Transport

**Figure 3:**
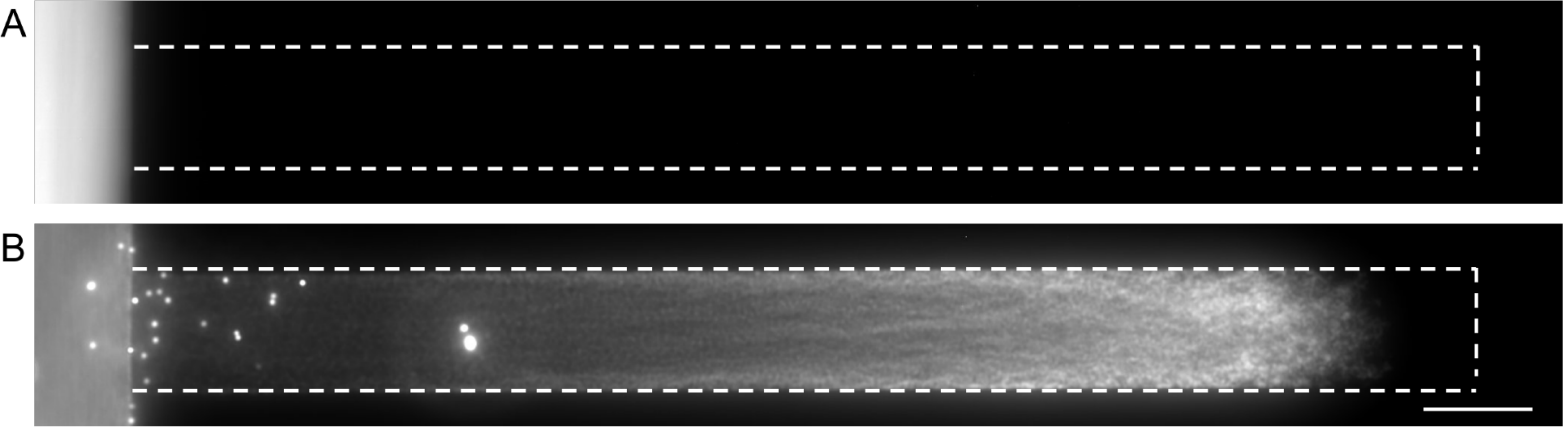
Microscope images of the dead-end channel. The dashed line represents the boundaries of the dead-end channel. The dead-end channel was filled with (A) MQ Water and (B) 10 mM NaCl, whereas the particle solution in the main channel contained MQ water and 0.05 mM NaCl, respectively. The microscope images are combined images obtained between *t* = 0 s and *t* = 300 s. Scale bar = 50 μm.

Before performing the salt gradient experiments, we did a control experiment to check whether particle movement could also be affected by other factors. In the control experiment, the dead-end channel was filled with water, and an air bubble was passed through the main channel to eliminate any mixing in the main channel beforehand (see Materials and Methods for more information). The particle suspension was prepared in MQ water and subsequently passed through the main channel. As shown in Figure 3A, the particles did not enter the channel when both the main and dead-end channels contained MQ water.

As already reported in previous studies,^10,15,23,25,34,38^ particles start entering the dead-end channel due to diffusiophoresis and diffusio-osmosis when a salt concentration gradient is present (Figure 3B). These processes can be theoretically described by the mobility and the relative gradient terms (eq 1). We also performed another control experiment with a salt gradient to check the effect of the main channel flow rate on the particle velocity in the dead-end channel (Supporting Information S6). The main channel flow rate was changed between 0.25 and 2 μL/min (70–560 μm/s), and it does not influence the particle velocity in the dead-end channel.

Two main factors influence the particle dynamics in the dead-end channel: (1) diffusiophoresis of the particles and (2) diffusio-osmotic flow generated by the channel walls. In our experimental setup, diffusiophoresis causes particles to move into the dead-end channel. Diffusio-osmotic flow on the wall induces a convective flow in the dead-end channel. Both scale with the relative gradient and corresponding particle and wall mobility. This leads to fluid circulation inside the channel to ensure continuity, where fluid near the wall is directed toward the channel entrance and fluid in the center toward the dead end. The summation of these two effects describes the velocity of the particle (*u*_p_ = *u*_DF_ + *u*), which is evidently a strong function of the particle position inside the dead-end channel.

**Figure 4:**
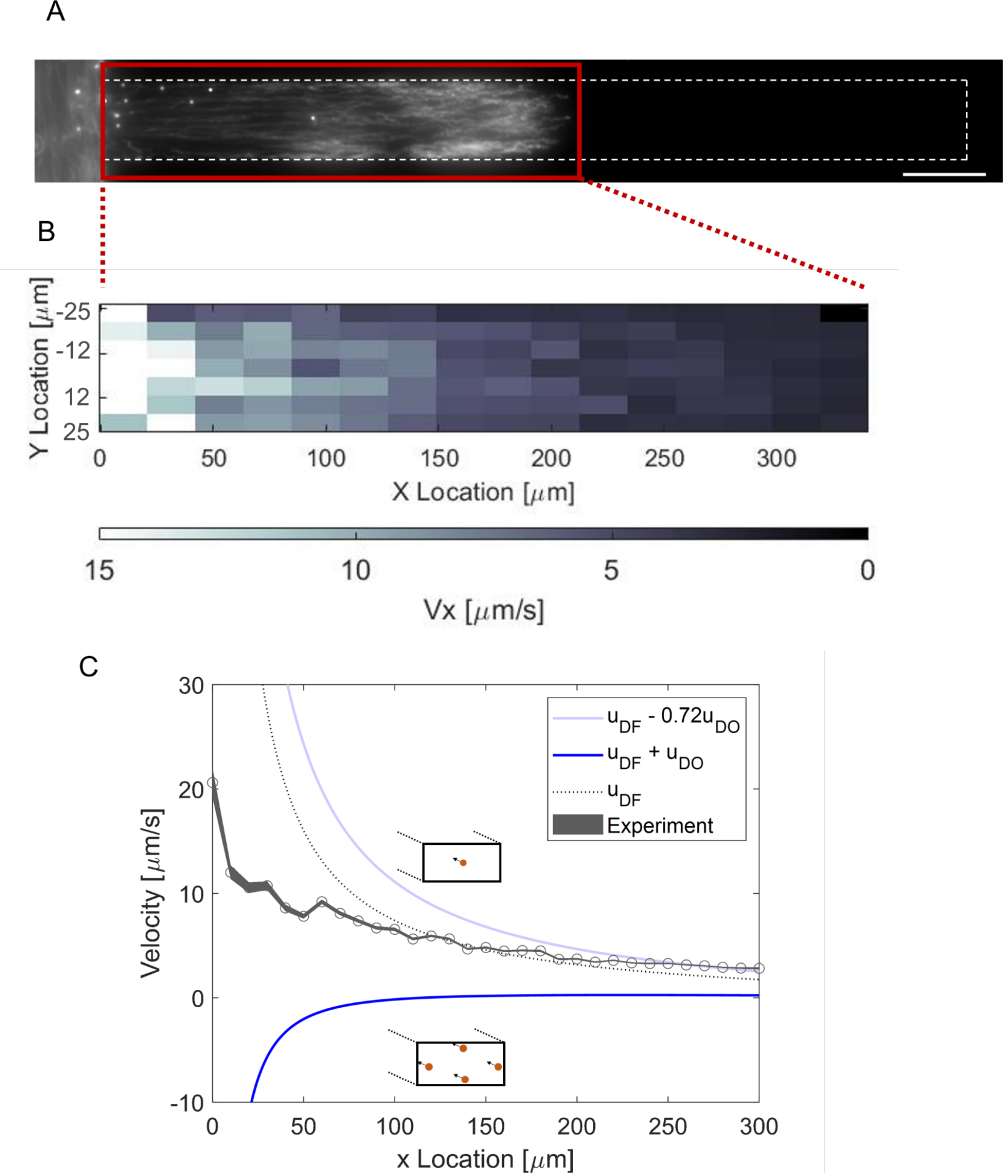
PS-carboxylate particles inside the dead-end channel, which was filled with 10 mM NaCl, and particle solution in the main channel containing 0.05 mM NaCl. (A) Stacked images of particles between 55 and 65 s. (B) 2-D average velocity profile calculated from particle tracked time interval 55 s < *t* < 60 s. (C) 1-D axial velocity calculated from particle tracking with time interval 55 s < *t* < 60 s. The black dotted line indicates the predicted velocity considering diffusiophoresis only. The light blue line represents the particle diffusiophoresis and centerline velocity due to diffusio-osmosis by the wall (*u*_upper_ = *u*_DF_ – 0.72*u*_slip_). The dark blue line shows the particle diffusiophoresis, and slip velocity (*u*_lower_ = *u*_DF_ + *u*_slip_). Scale bar = 50 μm. The shadow area represents the standard error (according to *N*_p_) of particle velocity.

Previous studies experimentally analyzed particle dynamics to understand the parameters and flow patterns based on the particle distribution across the dead-end channel.^15,38^ Here, we analyze these dynamics by calculating the average particle velocities in the dead-end channel (Figure 4). Figure 4A shows the overall movement of the particles for 10 s intervals, in which the average particle velocity was determined. The averaged velocity component in the *x* direction was obtained (Figure 4B) for the indicated *xy* plane. It reveals the gradual decrease in the particle velocity in the *x* direction, as well as velocity variations in the *y* direction. This behavior results from the relative gradient decrease and eventually results in particle accumulation toward the end of the dead-end channel. Additionally, we also observed that the average particle velocity is higher in the middle of the channel than near the sidewalls due to the opposite osmotic fluid flow.

We analyzed the average particle *x*-component velocity (Figure 4C) by averaging particle velocity in 10 μm windows (see Materials and Methods for the calculation procedure). We compare our result with the theoretical diffusiophoretic velocity description only (black dashed line). There are two regions where the theoretical diffusiophoretic velocity differs from the average experimentally estimated particle velocity. It underestimates the particle velocity near the entrance (<60 μm) and overestimates it further into the channel (>150 μm). Particle diffusiophores alone cannot capture the particle dynamics inside the dead-end channel. When we compare our experimental results against that including the effect of osmotic fluid flow (Supporting Information Figure S11A), the averaged velocity data fall within this range (in between light and dark blue lines). The ranges in Figure 4C were calculated based on the diffusiophoretic velocity and the osmotic convective flow. The upper range (light blue line) was determined with the *u*_upper_ = *u*_DF_ – 0.72*u*_slip_ equation (more information about the origin of the factor 0.72 can be found in Supporting Information Figure S9) and describes the maximum particle velocity in the middle of the channel where convective flow and diffusiophoresis are in the same direction. The bottom range (dark blue line) was determined with the *u*_lower_ = *u*_DF_ + *u*_slip_ equation and shows the particle velocity near the wall where convective flow is in the opposite direction of the diffusiophoresis. Figure 4C indicates that the experimental velocity is inside the range, and the averaged particle velocity is not following the theoretical particle diffusiophoretic velocity only.

We also determined the particle *y*-component velocity to analyze lateral movement. Kar et al.^23^ report a transverse drift of particles toward the side wall in the dead-end pore for NaCl in the first 250 μm. The theoretical particle velocity profile (Supporting Information Figure S11) shows that the particles tend to move toward the sidewall due to the circulating convective flow. Our *y*-component velocity analysis (Supporting Information Figure S7) revealed that particles move toward the sidewalls mostly in the first 20 μm of the channel entrance. Our observations are distinct from those of Kar et al.^23^ The difference likely originates from the different channel width which affects the circulating flow profile. Moreover, Kar et al.^23^ did not observe any lateral movement of particles in a KCl gradient. The solute type,^23^ as well as the dead-end channel width, can influence the lateral particle movement.

### Concentration-Dependent Zeta Potential Values for Diffusiophoresis and Osmosis

The zeta potential of the particle and wall is affected by the NaCl concentration. We have experimentally analyzed the particle and wall zeta potential concentration dependency. To this end, first, we measured particle (PS with carboxylate and RhB-PEG groups) zeta potential in the 1–10 mM NaCl range, where particles showed concentration dependency because of their surface groups (Supporting Information Figure S1). The absolute zeta potential value |ζ| decreases as the NaCl concentration increases since there is more ion accumulation in the electrical double layer.^50^ The zeta potential values were fitted by using ζ(*c*) = *a* + *b* log_10_*c*_NaCl_; as for |ζ| > 25 mV, the zeta potential value scales with log_10_(*cZ*^2^) according to the Gouy–Chapman–Stern double layer model.^19^ We also measured the streaming potential for PDMS surfaces to estimate the zeta potential, which is shown in Supporting Information Figure S3. The streaming potential of PDMS showed a strong dependency on the NaCl concentration. We checked our measurements with the available zeta potential values for PDMS, and our measurements agree well with the values provided in Kirby and Hasselbrink.^19^

In the literature, often an averaged zeta potential^15^ or the one at the highest concentration^25^ is used to predict the particle dynamics. We analyzed the effect of using a concentration-dependent zeta potential for both the particle and the wall.

**Figure 5:**
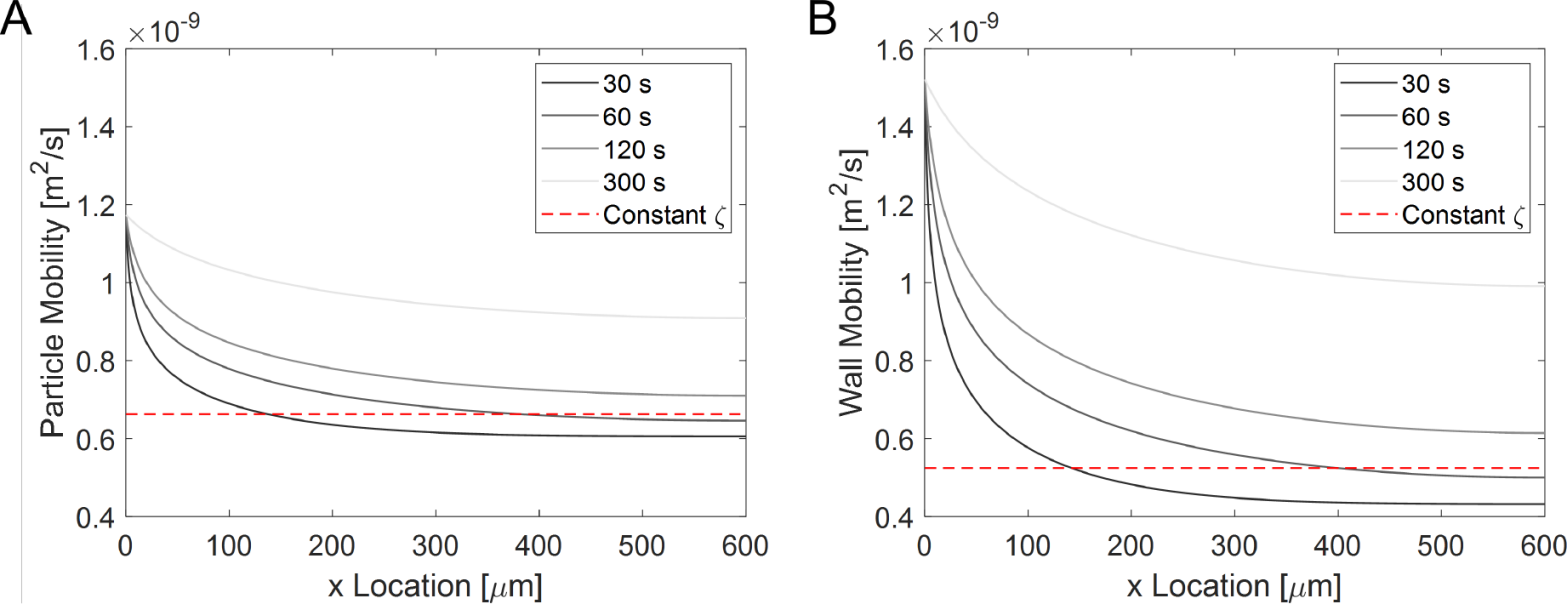
Theoretical mobility values for (A) PS particle with carboxylate groups and (B) PDMS wall. Each figure contains the concentration-dependent zeta potential (see Figures S1 and S3) mobility values at *t* = 30 s, *t* = 60 s, *t* = 120 s, and *t* = 300 s and the mobility value with a constant zeta potential value at 5 mM NaCl concentration (ζ_PS-carbox_ = −70 mV and ζ_PDMS_ = −60 mV).

The effect of concentration-dependent zeta potential on the local particle and wall mobilities is shown in Figure 5. The zeta potential directly influences the diffusiophoretic/diffusio-osmotic mobilities, and thereby particle velocity. The electrolyte-dependent particle and wall mobilities are higher at the entrance of the dead-end channel and approach a constant mobility further into the channel. The constant mobilities were calculated by using constant zeta potential value (for the wall (ζ_PDMS_ = −60 mV) and the particle ((ζ_PS-carbox_ = −70 mV)). Lower salt concentration leads to higher zeta potential, which results in higher mobilities that become evident at longer experimental times. The difference between the mobilities is substantial near the entrance for both cases and grows further in time.

We performed particle dynamic simulations to explore and understand the zeta potential dependency. Previous studies theoretically described the particle dynamics with a convection-diffusion equation, assuming constant zeta potentials.^10,15,34^ Here, we solved the same equations in 3-D and included concentration-dependent zeta potential values.

**Figure 6:**
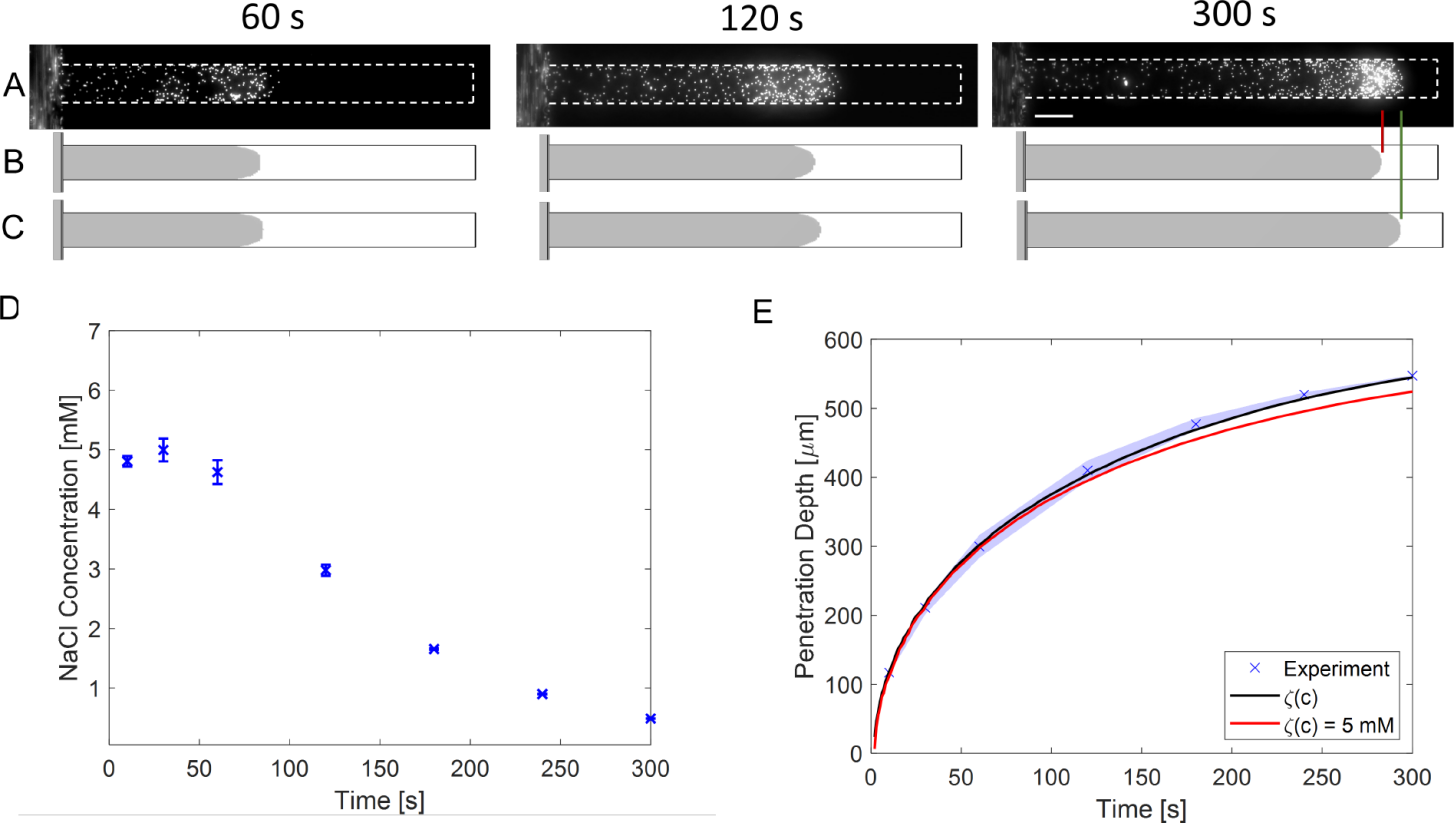
Experimental and simulation results for the PS particle with carboxylate groups. (A) The experimental profiles at *t* = 60 s, *t* = 120 s, and *t* = 300 s. Scale bar = 50 μm. Simulation assumes (B) a constant-zeta potential for the wall (ζ_PDMS_ = −60 mV) and the particles ((ζ_PS-carbox_ = −70 mV) and (C) concentration-dependent zeta potential values. The zeta potential fits and equations are given in the Supporting Information. The gray areas show the region where the particle concentration exceeds >0.1 and is used to track the front of the particles. (D) NaCl concentration versus time at the experimentally determined front particle position. (E) Experimental and simulation results of front particle position versus time. The error bars in D and the shadow area in E represent the standard deviation of three independent experiments.

**Figure 7:**
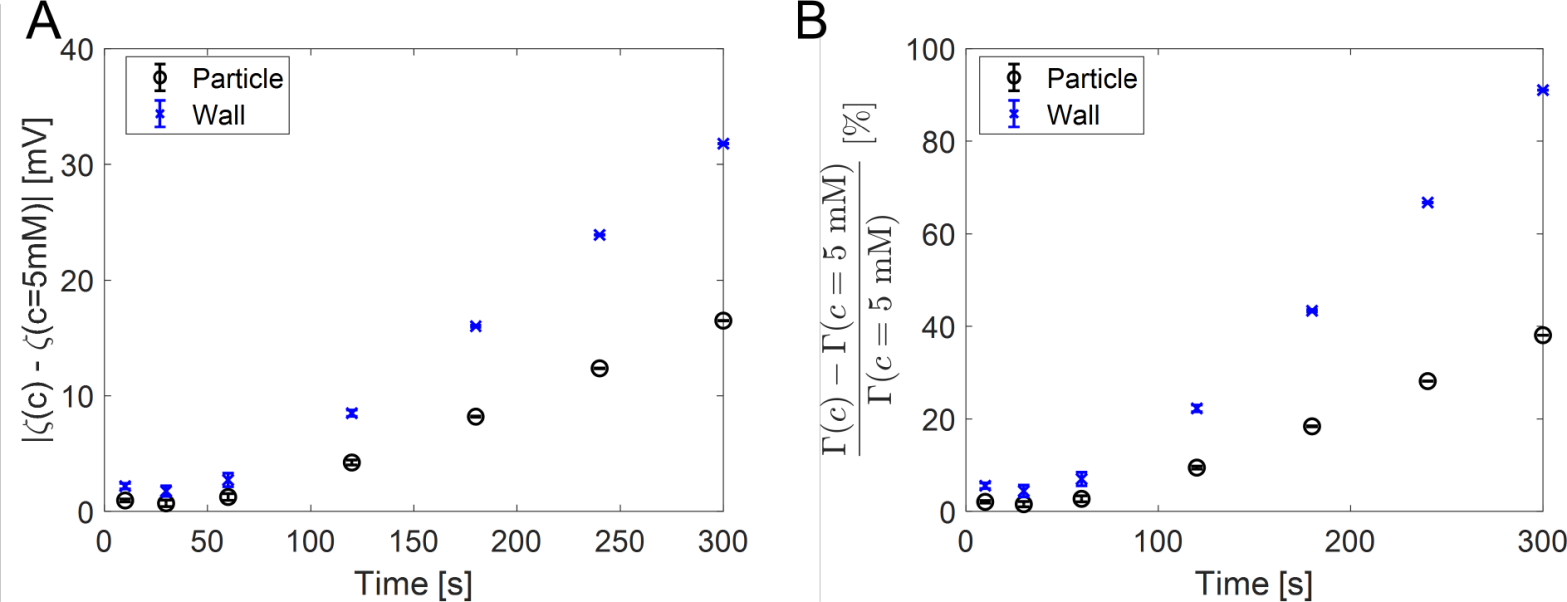
Effect of using concentration-dependent zeta potential. (A) Zeta potential difference between the average and the concentration-dependent zeta potential values at the front particle position. (B) Mobility difference between the average and concentration corrected mobility values at the front particle position. The error bars represent the standard deviation of three independent experiments.

Figure 6 shows the experimental results with the theoretical predictions at *t* = 60, 120, and 300 s. The wall and particle zeta potential values were included in the simulations using constant (for the wall (ζ_PDMS_ = −60 mV) and the particle ((ζ_PS-carbox_ = −70 mV)) as well as concentration-dependent zeta potential values in Figure 6B and C, respectively. Even though concentration-dependent zeta potential values for the particle and the wall were used, both simulation results aligned with the experimental observation until 120 s (see Video S1). We observed a difference between the two simulations for longer times, in line with the increasing difference in mobility as discussed before (Figure 5). Moreover, the change in mobility can also be explained by NaCl concentration at the experimental particle front position. As can be seen in Figure 6D, the NaCl concentration strongly reduces at the particle front position, which can affect the particle zeta potential significantly. However, as can be seen in Figure 6E, the particle front position is hardly affected. The particle mobility, however, is strongly affected by the local salt concentration (Figure 7B). Although the mobility is significantly affected by the local salt concentration, the effect on the particle front position is small due to the low local salt concentration gradient and consequently low particle diffusiophoretic velocity. It is also important to underline that the centerline fluid velocity becomes <0.5 μm/s (Supporting Information S9), which also suggests a reducing effect by the wall-induced osmosis.

We quantify this behavior in Figure 7, which shows the zeta potential and mobility differences compared to their constant values. The values were determined between the constant and the concentration-dependent zeta potential values at the front particle position in time. The difference becomes more than 10 mV after 120 s since the solute concentration becomes low. This further influences the diffusiophoresis and diffusio-osmosis mobilities and thereby particle velocity. Figure 7B displays the relative change in mobility values at that position. Using the constant zeta potential causes a much lower mobility prediction at longer times. Therefore, adjusting the zeta potential value according to the environment is essential. We also note that we obtain the largest zeta potential deviations in regions where the relative gradient is the lowest, hence reducing its effect on the particle dynamics.

**Figure 8:**
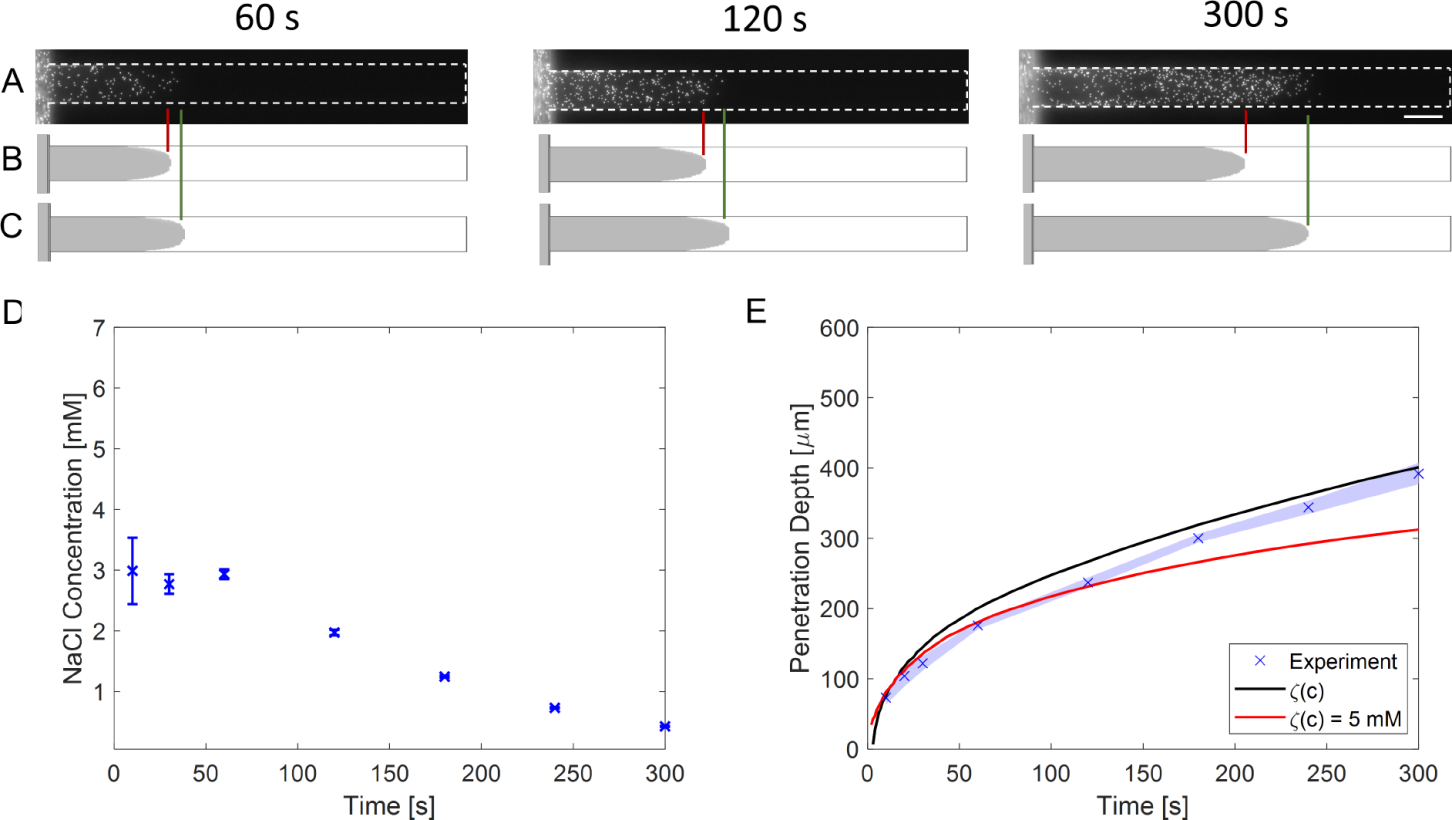
Experimental and simulation results for PEG-coated PS particles. (A) The experimental profiles at *t* = 60 s, *t* = 120 s, and *t* = 300 s. Scale bar = 50 μm. Simulation assuming (B) a constant-zeta potential for the wall (ζ_PDMS_ = −60 mV) and the particles (ζ_PS–PEG_ = −10 mV) and (C) concentration-dependent zeta potential values. The zeta potential fits and equations were given in the Supporting Information. The gray areas show the region where the particle concentration exceeds >0.1. (D) NaCl concentration versus time according to the experimentally determined front particle position. (E) Experimental and simulation results of front particle position versus time. The error bars in D and the shadow area in E represent the standard deviation of three independent experiments.

We repeated the experiments and simulations for PEG surface coated PS particles, where the particle’s zeta potential changes more strongly with the NaCl concentration since *b* in the ζ(*c*) equation is higher compared to uncoated particles (Supporting Information Figure S1). Figure 8 shows the experimental and simulation results at *t* = 60, 120, and 300 s. PS-PEG particles penetrate the dead-end channel less compared to uncoated particles since the zeta potential is ∼50–60 mV lower (Supporting Information Figure S1), causing a lower diffusiophoretic mobility. Second, we observed that the particle front has a strongly curved parabolic shape, caused by the significant diffusio-osmotic flow. Although the wall diffusio-osmosis is equal for both experiments, its influence on the resulting particle transport is different for each ζ_p_. Finally, we investigated the effect of concentration-dependent zeta potential on the simulation results in Figure 8B,C. We found that the concentration-independent simulations resulted in significantly lower particle infusion (see Video S2). The simulation results using the concentration-dependent zeta potential fit well with the experimental observations. In Figure 8E, the difference between the experimental particle position and the simulation result with constant zeta potential increases. Figure 8D shows the NaCl concentration at the experimental front particle position with time. Due to the overall lower diffusiophoretic mobility of PS–PEG compared to PS-carboxylate particles, the front is located in lower salt concentration but higher relative salt concentration gradients. Moreover, the centerline fluid velocity is around 1–2 μm/s (see Supporting Information), which is still significant compared to the particle’s phoretic velocity. This results in a significant difference between using a constant or concentration-dependent zeta potential.

We also checked experimentally whether all particle trajectories provide velocities that are inside the upper or lower boundaries (as given in Figure 4C). The individual particle trajectories are shown in Supporting Information Figure S8A,B. Some trajectories were not inside the range when constant zeta potential values for the particle and the wall were used, whereas trajectories were mostly inside the range when concentration-dependent zeta potential and mobility values were used. It is thus essential to use the concentration-dependent zeta potential and mobility value when one wants to describe all trajectories in the system.

## Conclusion

In this study, we investigated diffusiophoresis and diffusio-osmosis in a dead-end channel based on experimental and numerical studies. The solute concentration results show that the error between the 1-D and 3-D computations increases near the entrance of the dead-end channel due to convective flow circulation that is included in the 3-D simulation. The particle tracking and analysis approach revealed that the convective flow is an essential parameter in particle dynamic behavior. We explored the concentration dependency of the zeta potential, a most crucial electrokinetic parameter that defines the diffusiophoresis and diffusio-osmosis strength. We did not observe a significant difference between simulations and experimental observations for shorter times (<120 s). However, for longer times (>120 s), the concentration-dependent zeta potential value and the constant value started to deviate from each other. Experimental observations were better described by the use of a concentration-dependent zeta potential. For PEG-coated particles, the difference between concentration-dependent and constant zeta potential was even more significant, due to the strong salt concentration dependency and the enhanced influence of diffusio-osmosis. This insight is highly relevant for applications in the zeta potential measurement via dead-end channels, particle sorting, or separation using diffusiophoresis and diffusio-osmosis. The inclusion of concentration dependent mobilities is expected to be most significant near depletion boundaries, where strong gradients are combined with low concentrations, where zeta potentials are affected the most.

## Supporting Information Available

Information about the zeta potential values for the wall and particles with their properties, diffusiophoretic and diffusio-osmotic flow profiles with centerline velocities, possible experimental error sources, and trajectories of all particles (PDF)
Video S1: Video of experimental observations compared with simulation results for PS particles with carboxylate groups; play speed is 10x (0–300 seconds); scale bar is 50 μm (MP4)
Video S2: Video of experimental observations compared with simulation results for PS particles with PEG groups; play speed is 10x (0–300 seconds); scale bar is 50 μm (MP4)
