# Supplementary material for: Diffusiophoresis and Diffusio-osmosis into a Dead-End Channel: Role of the Concentration-Dependence of Zeta Potential
Source: Langmuir. 2023 Jan 28;39(6):2322–32. doi: 10.1021/acs.langmuir.2c03000 (PMC9933534; doi:10.1021/acs.langmuir.2c03000)
Supplement: Supplementary file 1 — la2c03000_si_001.pdf [file la2c03000_si_001.pdf]

# Supporting Information: Diffusiophoresis & Diffusio-osmosis into a Dead-End Channel: Role of the Concentration-Dependence of Zeta Potential

Burak Akdeniz, Jeffery A. Wood,* and Rob G. H. Lammertink*

*Soft Matter, Fluidics and Interfaces, University of Twente, MESA+ Institute for Nanotechnology, P.O. Box 217, 7500 AE Enschede, The Netherlands*

E-mail: j.a.wood@utwente.nl; r.g.h.lammertink@utwente.nl

Phone: +31 (0)534892961; +31 (0)534892063

## Contents

**Supporting Information Available** **3**

**1 Particle Properties** **3**

**2 Zeta Potential of Particles** **3**

**3 Effect of Gravity** **5**

**4 Zeta Potential of Polydimethylsiloxane (PDMS)** **5**

**5 Experimental Error Sources** **5**

**6 Cross sectional NaCl concentration profile** **8**

**7 Main Channel Flow Rate** **8**

**8 Particle Velocity – $y$ component** **10**

**9 All particles trajectories** **11**

**10 Surface-induced Flow** **11**

**11 PEG Zeta Potential and Wall Mobility Change** **13**

**12 Theoretical Velocity Profiles** **13**

**13 Centerline Velocities** **15**

**14 Video S1 and S2** **15**

**References** **15**

# Supporting Information Available

## 1 Particle Properties

Two particles were used in this study. The general information for each particle is given below.

Table S1: General properties of particles that were used in the experiments.

| Particle Surface | Diameter [µm] | Abs./Em. [nm] | $D_p \cdot 10^{13}$ [m$^2$/s] | Additive |
|---|---|---|---|---|
| Polyethylene glycol(PEG) | 1.09±0.04 | 560/584 | 3.99 | – |
| Carboxylate | 1.00±0.03 | 580/605 | 4.35 | + (2 mM Sodium Azide) |

## 2 Zeta Potential of Particles

The zeta potential was measured in the 1-10 mM NaCl range and given in Figure S1. The equation ($\zeta(c_{NaCl}) = a + b\log_{10}(c_{NaCl})$) was fitted for each particle's zeta potential value. The constants $a$ and $b$ were calculated as -81.61 mV and 15.61 mV for PS-carboxylate, and -27.43 mV and 22.63 mV for PS-PEG particles ($c_{NaCl}$ is in mM).

The zeta potential change depends on the solute relative gradient, and the $b' = b/2.3026$ values (EquationS2).

$$\frac{d\zeta}{dc} = \frac{b'}{c}, \tag{S1}$$

$$\frac{d\zeta}{dx} = b'\frac{d\ln c}{dx}, \tag{S2}$$

Since the solute relative gradient is the same, $b'$ value determines the value of the zeta potential change. Figure S2 suggested that the zeta potential change is high at the dead-end channel entrance and reducing in time.

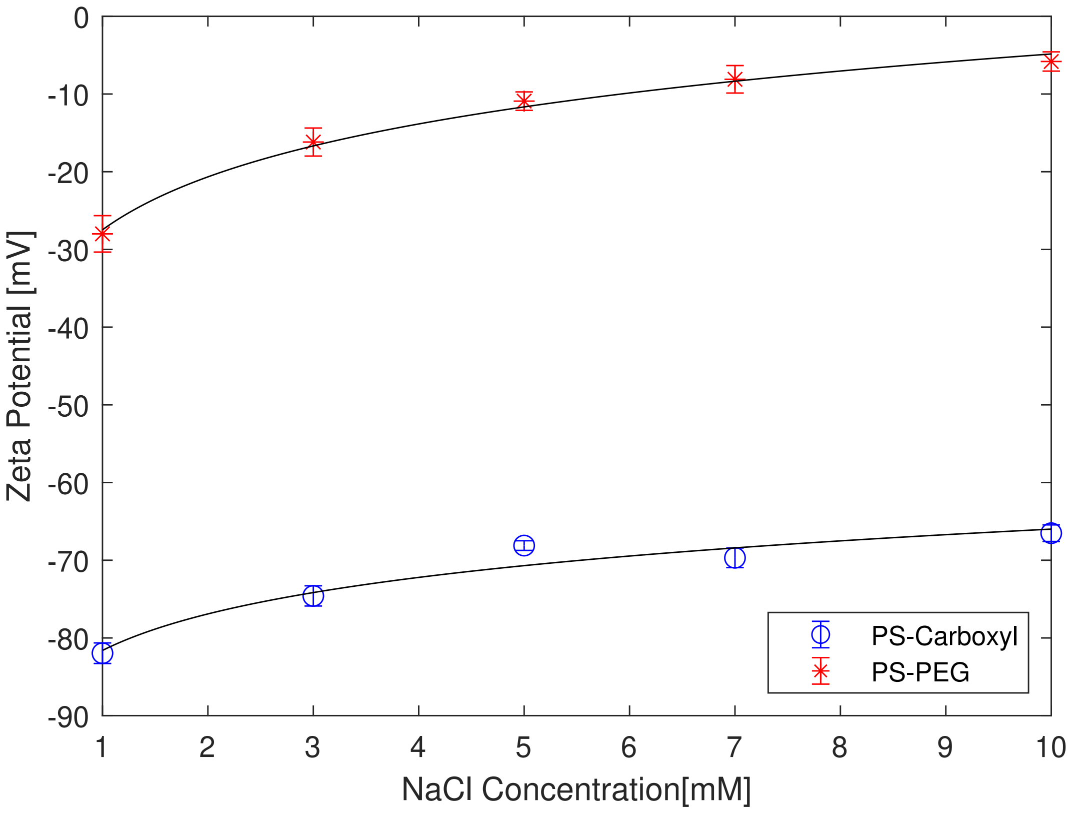


Figure S1: Zeta Potential of particles in NaCl solutions. Two different particles, PS-carboxylate, and PS-PEG were used in this study. The equation ($\zeta = a + b \log_{10} c_{NaCl}$)) was fitted for each particle's zeta potential value. The error bar shows the standard deviation of three samples.

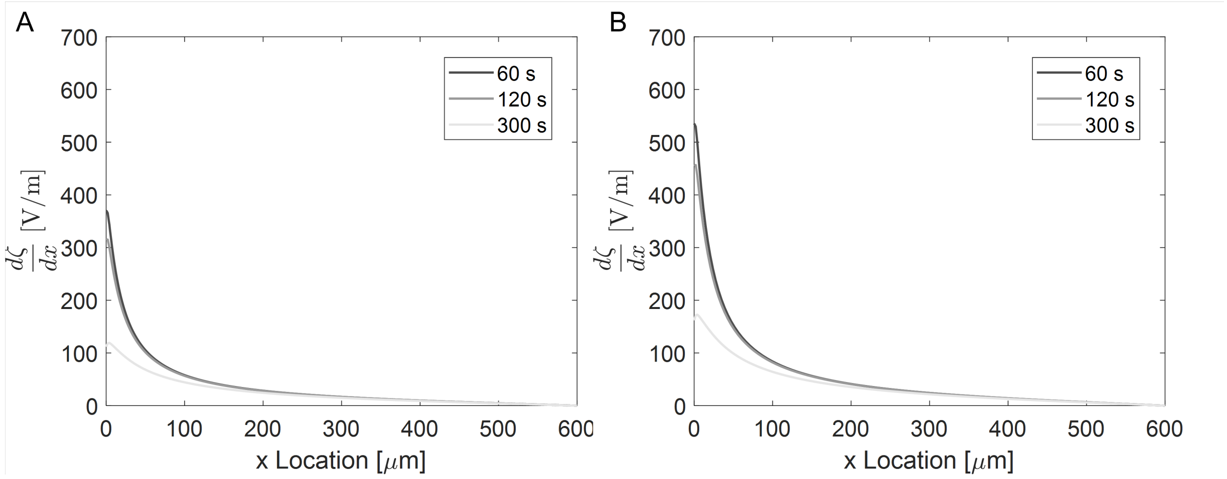


Figure S2: Zeta Potential change according to $x$ location. (A)PS-carboxylate, and (B)PS-PEG particles.

## 3 Effect of Gravity

The particles are significant in size ($\approx$ 1 µm) compared to the channel height (10 µm). Even though the density difference between the particle and water ($\Delta\rho = 50$ kg/m$^3$) is low, particles tend to move towards the bottom in time. When the particles start in the middle of the channel, they move to the bottom wall in about 2-3 minutes. However, the electrokinetic force and wall exclusion effect prevent particles from sticking to the bottom. Moreover, the confocal microscope images of similar size particles[1] show the effect of gravity is not extreme.

## 4 Zeta Potential of Polydimethylsiloxane (PDMS)

The zeta potential of PDMS was calculated by streaming potential measurements in 0.1 - 10 mM NaCl. The zeta potential results were compared with literature ($\zeta = a + b\log_{10}(c)$ with a=6.27 mV and b=29.75 mV).[2] A similar trend is observed (Figure S3). Values for a=6.27 mV and b=29.75 mV were employed in the simulations.

Figure S4 showed that the zeta potential change is high at the dead-end channel entrance and reducing in time.

## 5 Experimental Error Sources

There are three primary sources of error due to particle detection and calculation methods. Firstly, the particle position might not be detected precisely due to the finite size of pixels in the camera, and this uncertainty is estimated by.[3]

$$(\Delta V)^2 = \frac{(\Delta x_{j+1})^2 + (\Delta x_j)^2}{(\Delta t)^2} = 2\left(\frac{\Delta x}{\Delta t}\right)^2 \tag{S3}$$

where $\Delta V$ is velocity uncertainity, and $\Delta t$ is the image time frame. $\Delta x$ is the uncertainity position and estimated as micron to pixel ratio over how many pixels are used for detect a particle (= 0.327/7 µm). This source of error is inversely proportional to the image frame

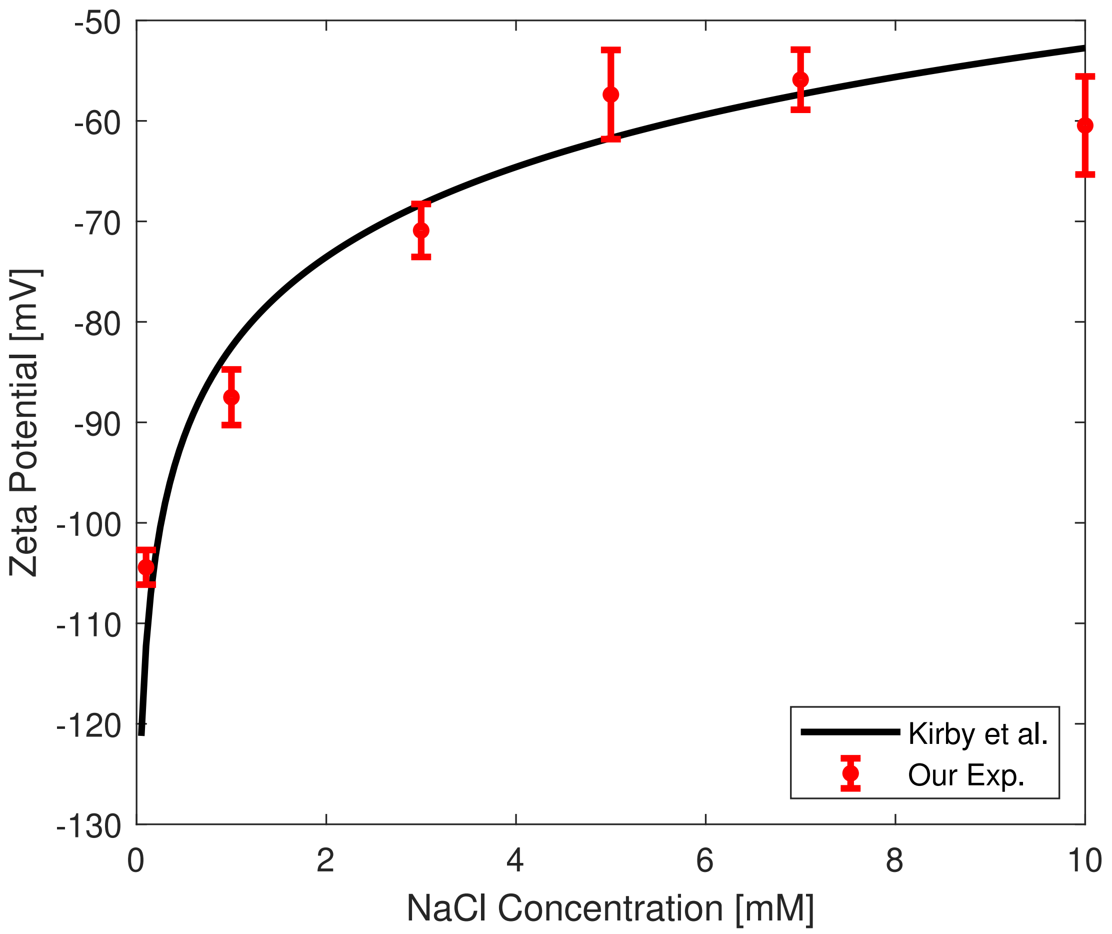


Figure S3: Zeta Potential of Polydimethylsiloxane (PDMS). The graph contains data from literature[2] and our experimental analysis.

rate and calculated for 10 fps as 0.66 μm/s, whereas for 1 fps as 0.06 μm/s. Therefore, this error source is relatively small.

Secondly, Brownian motion and the particle velocity might be of the same magnitude when the particle velocity is small (< 1 μm/s). The relative error is determined by the following formula that describes the relative error in the PIV system:[4]

$$\epsilon_x = \frac{1}{U}\sqrt{\frac{2D_p}{\Delta t}} \tag{S4}$$

where $U$ is the particle's velocity, and $D_p$ is the diffusivity of the particle. We determined

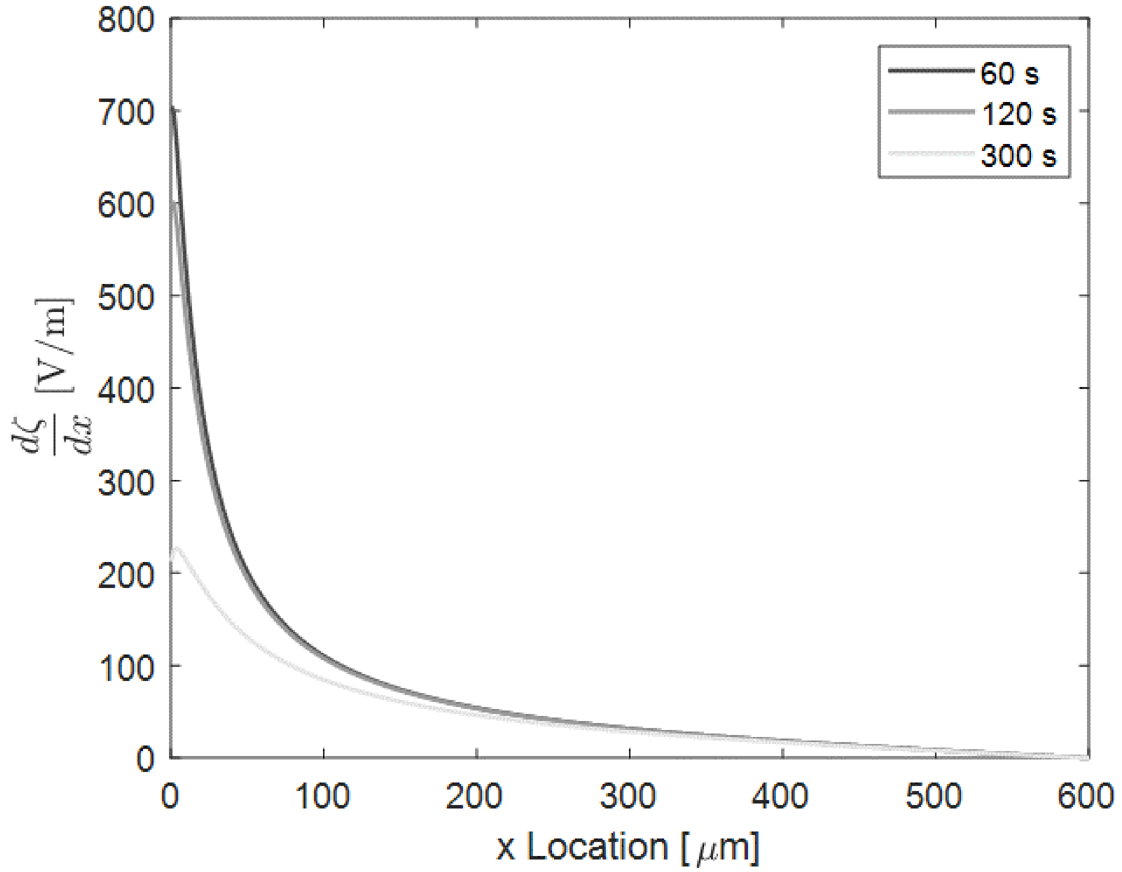


Figure S4: PDMS Zeta Potential Change according to $x$-Location.

the limit which is the error, and the velocity magnitudes are equal ($\epsilon_x = U$). These limits are 1.7 µm/s for 10 fps, and 0.96 µm/s for 1 fps. Velocity magnitudes below these limits might be due to Brownian motion.

Thirdly, the response of particles under sudden acceleration becomes important in low viscosity. The response time is inversely proportioned to environmental viscosity, and it is estimated by;[4]

$$\tau_p = d_p^2 \frac{\rho_p}{18\eta} \tag{S5}$$

The response time is 78 ns which is much lower than the image frame time. It is negligible in the analysis.

# 6 Cross sectional NaCl concentration profile

NaCl concentration profiles were calculated by the convective-diffusion equation for 1-D and the 3-D domains. The result of the concentration (Figure 2) underlines there is no significant difference in the concentration beyond ≈ 5 µm into the channel. The concentration profile does differ between 1-D and 3-D domains near the entrance of the dead-end channel. We suspect that the convective flow in the main channel causes this concentration difference. To visually show this effect, the cross-sectional ($y$-$z$ plane) NaCl concentration profile is given in Figure S5.

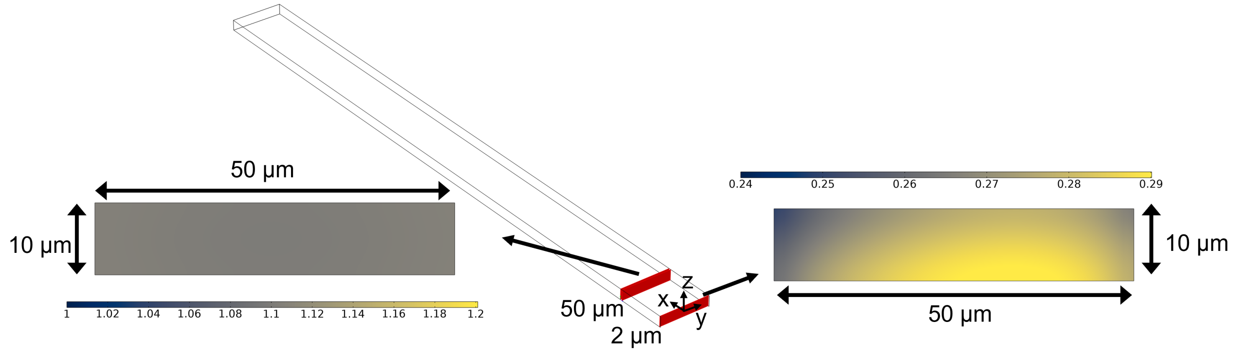


Figure S5: NaCl concentration profile was simulated in 3-D domain. $y$-$z$ cross plane concentration profile was given in 2 µm, and 50 µm $x$ location after 60 seconds. The color bar unit is mM.

# 7 Main Channel Flow Rate

The main channel flow rate (<2 µL/min) does not influence the particle velocity in the dead-end channel. Here, we performed experiments with four flow rates; 2 µL/min, 1 µL/min, 0.5 µL/min, and 0.25 µL/min (70 - 560 µm/s). The particle velocity analysis (Figure S6) shows that the velocity values are very similar.

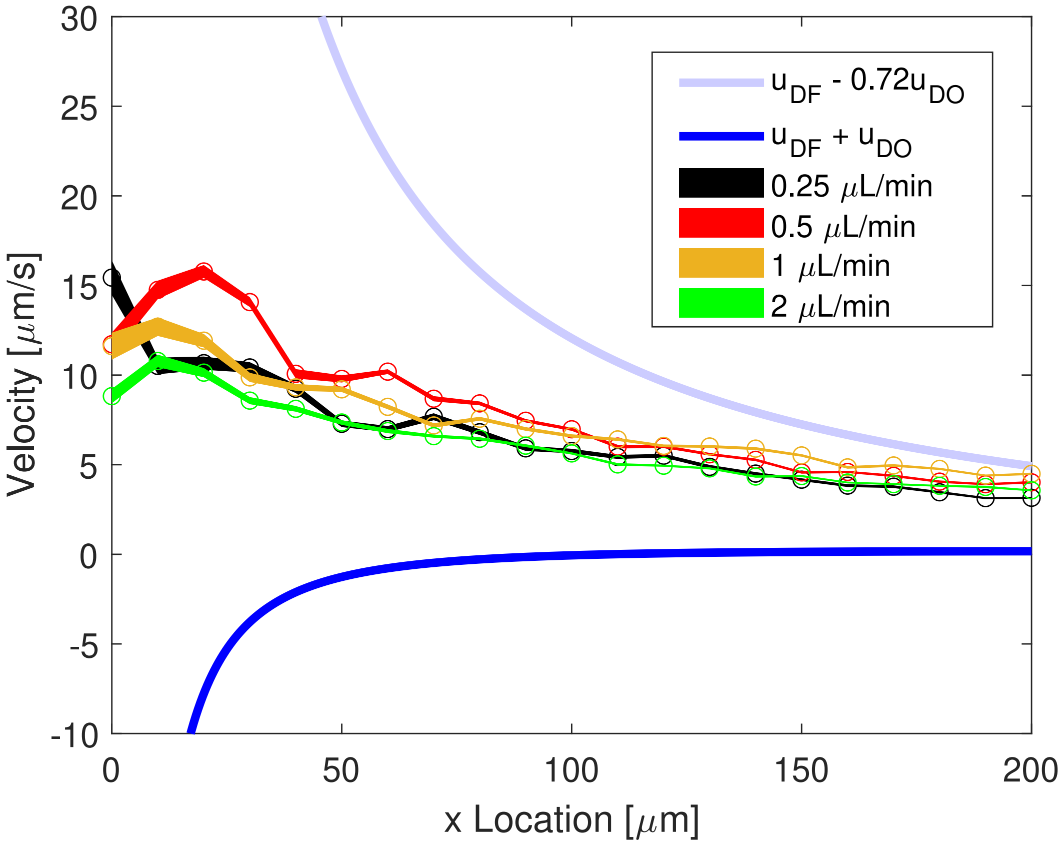


Figure S6: The main channel flow rate does not influence the particle velocity in the dead-end channel. The velocities of the particles are calculated at 60 s.

# 8 Particle Velocity – $y$ component

The highest movement in the particle is observed in the $x$-direction. $y$ component of the absolute velocity value shows particles do not perform a lateral movement (Figure S7). We only observed a relatively high-velocity profile at the entrance of the dead-end channel. After $\approx 20 >$ µm, we only observed an error in the particle velocity due to Brownian motion. Our error calculation for Brownian motion for the PIV case (for 5fps $\approx$ 1.15 µm/s) also supports this observation.

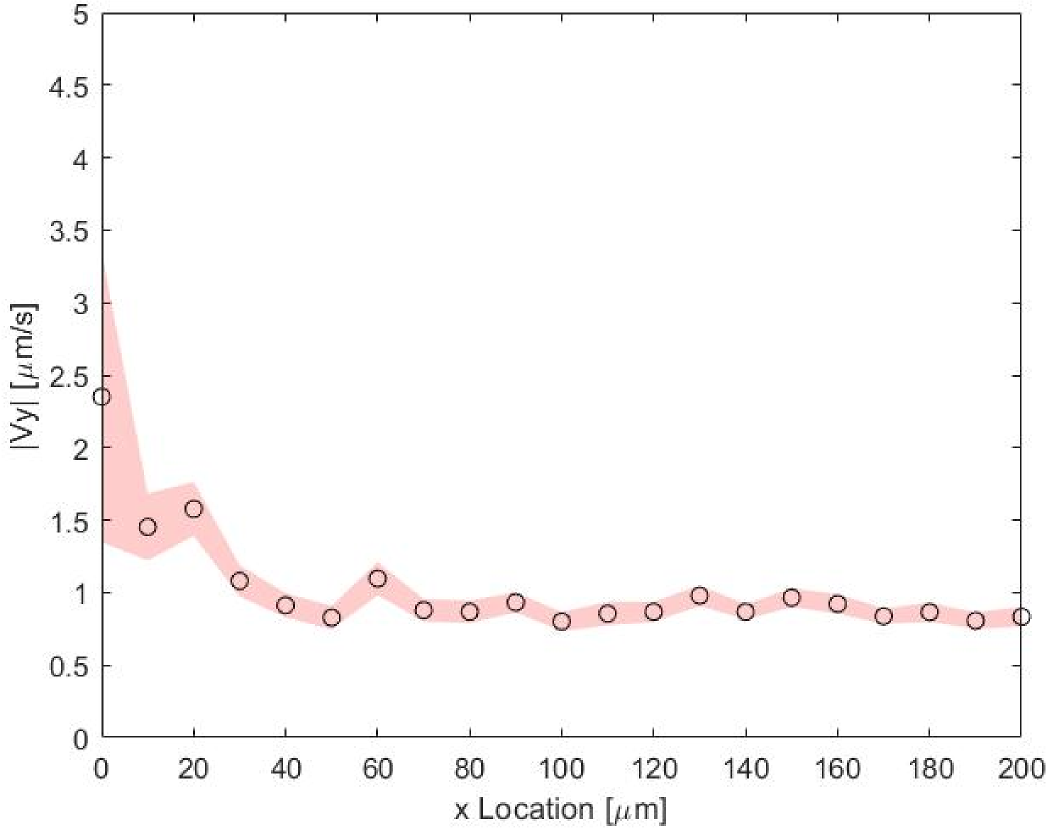


Figure S7: Absolute $y$-component of particle velocity for PS-carboxylate particle at 60 seconds. In the calculation of the particle velocities, we reduced the frame rate to 5fps. The enclosed area shows the standard error calculated according to the number of particles.

# 9 All particles trajectories

Approximately 300-400 particles were tracked in one experimental study. Figure 4 shows the average of those particles in certain locations in the 1-D domain. Each particle's $x$ component velocity value is given according to the $x$ direction. The range given in Figure S8 (light and dark blue lines) were calculated by combining the particle diffusiophoresis, and fluid flow and we performed calculations for concentration-independent (Figure S8A) and concentration-dependent zeta potential values(Figure S8B). Almost all the particle trajectories were captured when the concentration-dependent zeta potential values were used.

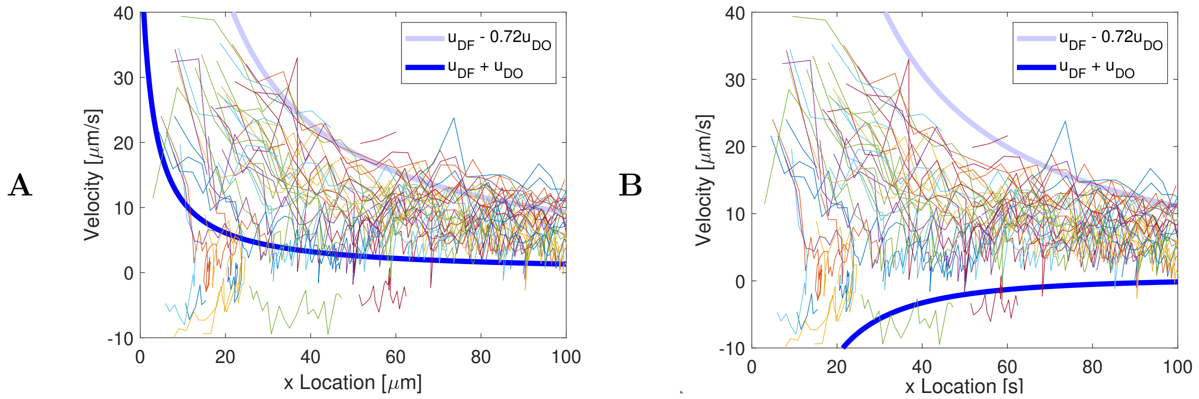


Figure S8: Particle $x$ component velocity in $x$-direction for PS-carboxylate particles at 60 seconds. In the calculation of range (light and dark blue lines) (A) concentration-independent and (B) concentration-dependent zeta potential values were used.

# 10 Surface-induced Flow

Surface-induced flow on the channel wall creates convective flow inside the dead-end channel. However, the magnitude of the center line convective velocity is not equal to the magnitude of the slip velocity ($u_{slip} \neq -u_{centerline}$). Figure S9 shows the $x$-component velocity profile at the $y$-$z$ plane. As can be seen from the figure, the velocity magnitude near the wall is different from the middle suggesting that the slip velocity is higher compared to the centerline velocity. We determined centerline velocity is $\approx 0.72$ of the slip velocity ($u_{slip} = -0.72 \cdot u_{centerline}$).

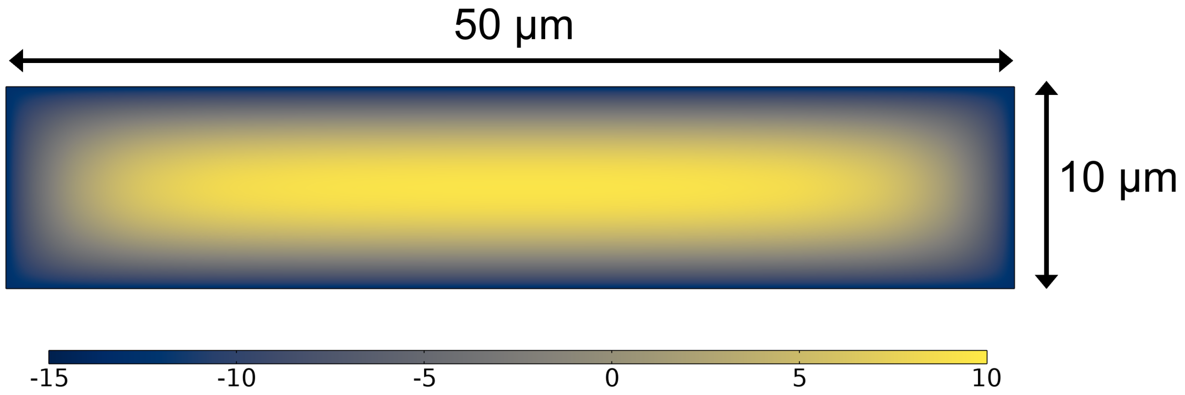


Figure S9: $y$-$z$ cross-plane velocity profile at $x$ = 50µm and t= 60 s.The color bar is given below the image, and the unit is µm/s.

# 11 PEG Zeta Potential and Wall Mobility Change

We performed the particle simulation for the constant zeta potential values for the wall ($\zeta_{PDMS} = -60mV$) and concentration-dependent zeta potential value for the PS-PEG particles. We also quantify behavior in Figure S10 which shows the zeta potential and mobility differences compared to their constant values.

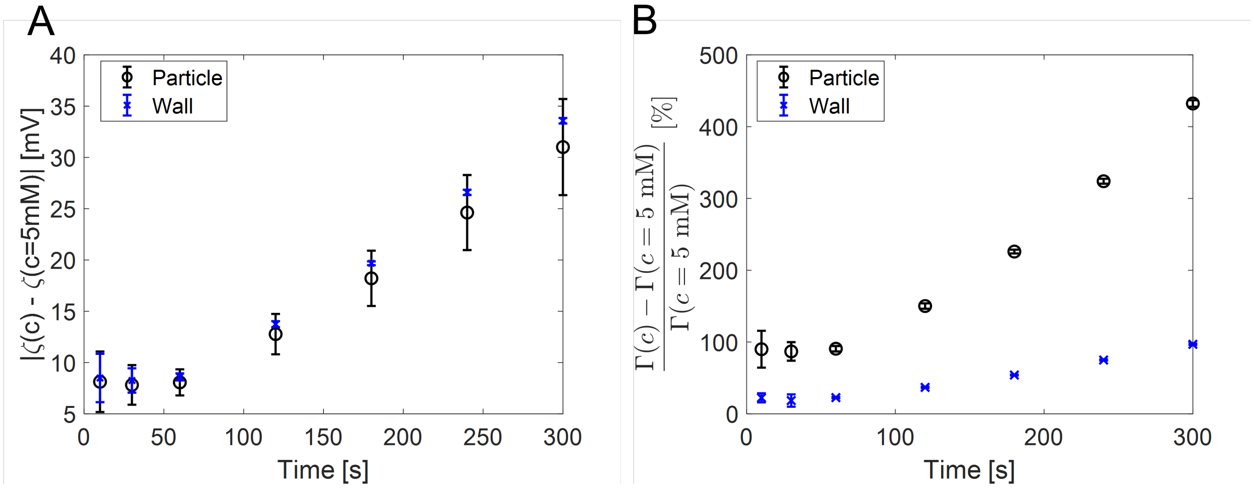


Figure S10: Effect of using concentration-dependent zeta potential. (A) Zeta potential difference between the constant $\zeta_{PS-PEG}$= -10 mV & $\zeta_{PDMS}$= -60 mV, and the zeta potential value where the front particle position. (B) Mobility difference between the average $\Gamma_{PS-PEG} = 4.59 \times 10^{-11}\ m^2/s$ & $\Gamma_{PDMS} = 5.24 \times 10^{-10}\ m^2/s$, and the mobility value where the front particle position.

# 12 Theoretical Velocity Profiles

We showed the theoretical velocity profile of convective flow and diffusiophoresis at 60 seconds below.

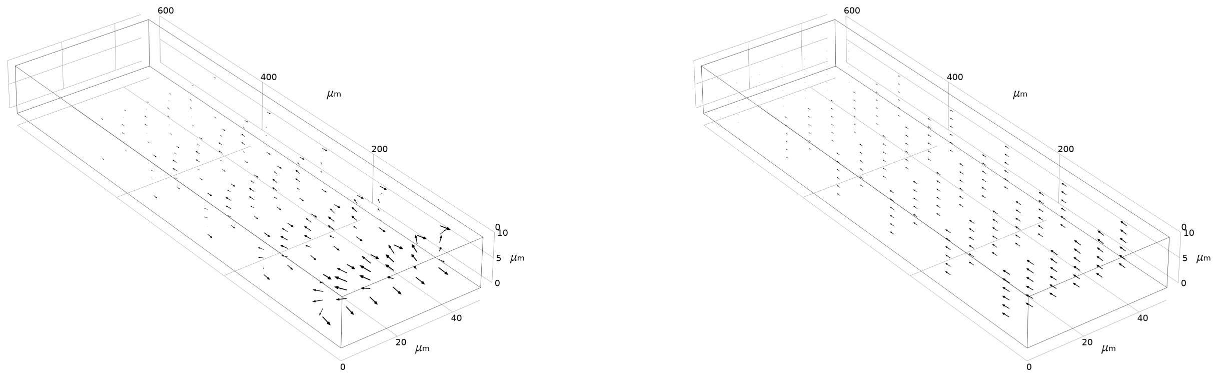


(A) Convective Flow

(B) Diffusiophoresis

Figure S11: Theoretical velocity profiles inside the dead-end channel. Arrows represent the directional velocity profile at t = 60 s. (A) Convective flow is present inside the dead-end pore due to slip velocity at the walls. It is induced by diffusio-osmosis flow at the wall. (B) Particle diffusiophoretic velocity profile. It scales with the relative gradient. Particles velocity in the dead-end channel is defined as convective flow (A) + diffusiophoresis (B).

# 13 Centerline Velocities

We calculated the centerline velocity according to front particle position by considering constant and concentration-dependent zeta potential cases.

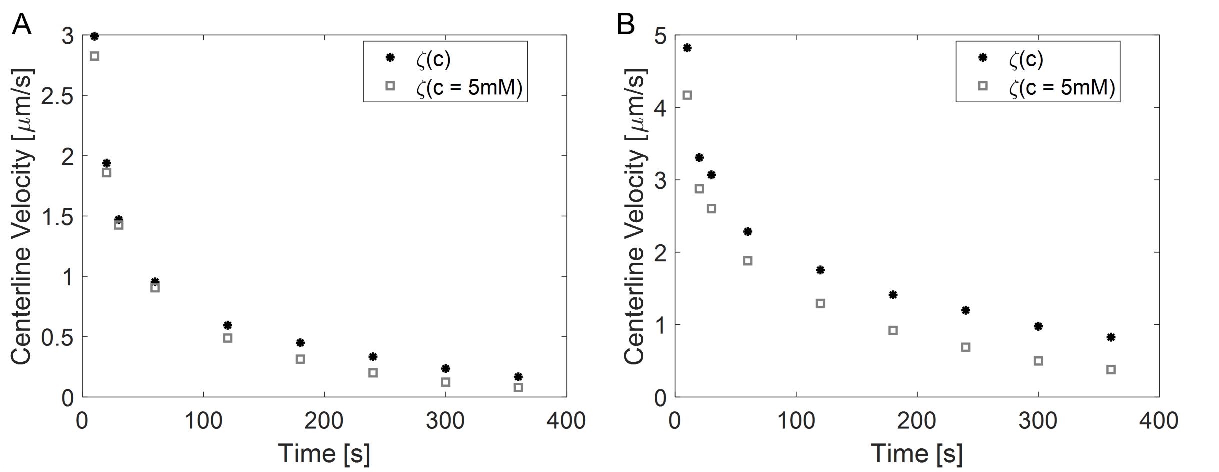


Figure S12: Centerline velocities of the (A)PS-carbox and (B)PS-PEG cases. The centerline velocity is calculated by the front particle location.

# 14 Video S1 and S2

Video S1 is the video of Figure 6 A,B and C. Video S2 is the video of Figure 8 A,B and C. For each video, play speed is 10x (0-300 seconds) and scale bar is 50 µm.

# References

(1) Shin, S.; Ault, J. T.; Feng, J.; Warren, P. B.; Stone, H. A. Low-Cost Zeta Potentiometry Using Solute Gradients. *Adv. Mater.* **2017**, *29*, 1701516.

(2) Kirby, B. J.; Hasselbrink, E. F. Zeta potential of microfluidic substrates: 2. Data for polymers. *Electrophoresis* **2004**, *25*, 203–213.

(3) Feng, Y.; Goree, J.; Liu, B. Errors in particle tracking velocimetry with high-speed cameras. *Rev. Sci. Instrum.* **2011**, *82*, 1–7.

(4) Raffel, M.; Kähler, C.; Willert, C.; Wereley, S.; Scarano, F.; Kompenhans, J. *Particle Image Velocimetry A Practical Guide*, 3rd ed.; Springer: Germany, 2018; Vol. 2.
